# Shaping of Innate Immune Response by Fatty Acid Metabolite Palmitate

**DOI:** 10.3390/cells8121633

**Published:** 2019-12-13

**Authors:** Hong-Tai Tzeng, I-Tsu Chyuan, Wei-Yu Chen

**Affiliations:** 1 Institute for Translational Research in Biomedicine, Kaohsiung Chang Gung Memorial Hospital, Kaohsiung City 83301, Taiwan; htay11@cgmh.org.tw; 2 Department of Internal Medicine, Cathay General Hospital, Taipei 10630, Taiwan; itchyuan@gmail.com; 3 Department of Medical Research, Cathay General Hospital, Taipei 10630, Taiwan; 4 School of Medicine, College of Medicine, Fu Jen Catholic University, New Taipei City 24205, Taiwan

**Keywords:** fatty acid metabolism, innate cells, pattern recognition receptor, palmitate, inflammation, pathogenesis

## Abstract

Innate immune cells monitor invading pathogens and pose the first-line inflammatory response to coordinate with adaptive immunity for infection removal. Innate immunity also plays pivotal roles in injury-induced tissue remodeling and the maintenance of tissue homeostasis in physiological and pathological conditions. Lipid metabolites are emerging as the key players in the regulation of innate immune responses, and recent work has highlighted the importance of the lipid metabolite palmitate as an essential component in this regulation. Palmitate modulates innate immunity not only by regulating the activation of pattern recognition receptors in local innate immune cells, but also via coordinating immunological activity in inflammatory tissues. Moreover, protein palmitoylation controls various cellular physiological processes. Herein, we review the updated evidence that palmitate catabolism contributes to innate immune cell-mediated inflammatory processes that result in immunometabolic disorders.

## 1. Introduction

Lipid species are the major and most abundant constituents of living systems. The essential plasma membrane components comprise lipid species such as glycerophospholipids, sphingolipids, and sterol lipids [1]. In addition to serving as the fundamental units of the cell membrane, lipids are energy sources involved in signal transduction pathways for cellular homeostasis maintenance, and thus regulate cell behaviors in response to stress or environmental stimuli [2,3]. Several studies have demonstrated links between fatty acid transporters, lipid metabolic reprogramming, and disease progression [4,5,6]. Dysregulation of lipid metabolism has been implicated in many diseases, such as metabolic disorders, cancer, atherosclerosis, cardiovascular disease, infections, and inflammatory diseases.

It has been shown that lipid metabolites contribute to the regulation of host immune systems. Bioactive lipid mediators govern a wide scope of immune responses, which involve a variety of inflammatory processes and coordinate immune cell functions, leading to pathogenesis of metabolic-related disorders. Among them, palmitate plays a predominant role in immune cell network coordination. In this review, we focus on the regulatory roles of the fatty acid palmitate in controlling immune balance. We also discuss how palmitate modulates and contributes to the effector function of innate immune cells, which in turn mediates pathogenic inflammation.

## 2. Fatty Acid Metabolism is Linked to Diseases

### 2.1. Fatty Acid Metabolic Pathways

Fatty acid β-oxidation is a major process that provides energy by degrading fatty acids. The enzymes responsible for fatty acid β-oxidation are mainly located in the mitochondria and peroxisomes. Each β-oxidation cycle can generate one acetyl-CoA molecule, which serves as the source for tricarboxylic acid cycle progression and yields more ATP per carbon than that from sugars by oxidative phosphorylation [7]. In contrast to the removal of two carbons from long-chain fatty acids in each β-oxidation round, fatty acid synthesis is catalyzed by joining two carbon units to the growing acyl chain via the cytosolic fatty acid synthase complex [8]. Palmitate is a 16-carbon saturated fatty acid produced via the fatty acid synthesis pathway in a fatty acid synthase-dependent manner, and acts as the major lipid mediator in inflammatory response regulation.

### 2.2. Palmitic Acid-Regulated Innate Immune Responses

**Figure 1:**
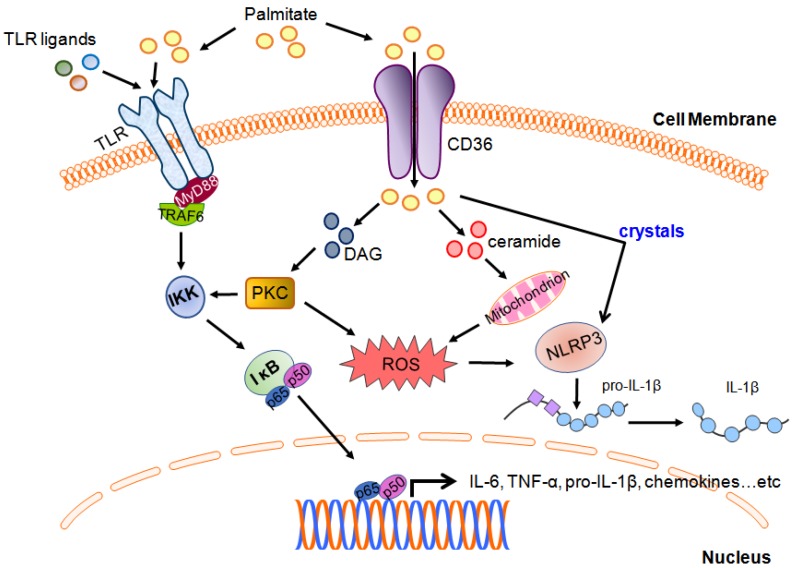
Schematic diagram showing the palmitate-mediated pathways and interplay with innate receptor signaling. Palmitate uptake by CD36 leads to the accumulation of deleterious lipids, such as diacylglycerol (DAG) and ceramides. DAG subsequently activates protein kinase C (PKC), which in turn leads to NF-κB activation via I kappa B kinase (IKK) activity. IKK is also activated by TRAF6, the downstream effector of the TLR/MyD88 axis. Additionally, the increase in ceramides resulting from palmitate uptake induces reactive oxygen species (ROS) production through mitochondrion activation, leading to interleukin (IL)-1β generation by nod-like receptor containing a pyrin domain (NLRP3) inflammasome. The intracellular crystallization of palmitate also induces NLRP3-mediated IL-1β release [18].

Cell uptake of palmitate is mainly mediated by the membrane fatty acid transporter CD36, also known as scavenger receptor B2 [9], although palmitate can also enter cells by other mechanisms, including direct membrane interactions, albumin-mediated transfer, and lipoprotein lipase-mediated uptake [10]. Accumulating evidence suggests a critical role by palmitic acid in modulating the activation of pattern recognition receptors in innate immune cells (Figure 1). Indeed, accumulating evidence reveals that although not through direct engagement of Toll-like receptors (TLRs), palmitate can modulate TLR downstream signaling in response to their ligand stimulation [11,12]. Palmitate is believed to be a TLR4 agonist that promotes macrophage activation and lipid metabolism-associated inflammation. Recently, however, it has been demonstrated that palmitate-induced inflammatory effects are not triggered by the direct binding of palmitate to TLR4, but through a combination of palmitate-mediated and TLR4-dependent priming, which alters cellular lipid metabolic pathways, gene expression, and membrane lipid composition, the prerequisite changes that are sufficient for palmitate-induced inflammation [12]. Specifically, TLR4-dependent priming alters cellular lipidome and membrane lipid composition; both changes are required for the sensitization of palmitate-induced inflammation. In addition, palmitate can modulate TLR4-mediated cellular response through induction of JNK activation [12]. These findings indicate that palmitate is not a bona fide agonist for TLR4. Previous consensus considers that palmitate acts as the agonist for TLR4 mainly due to contamination of bovine serum albumin (BSA), as combination treatment of BSA preparations with LPS can account for palmitate-induced TLR4 activation [11]. However, pharmacological inhibition of TLR4 abrogates LPS-triggered but not palmitate-induced JNK activation, which suggests that two independent processes are involved in mediating the LPS- and palmitate-induced pathway [12]. Mogilenko et al. reported that a palmitate-enriched environment amplified TLR-mediated responses by suppressing TLR-induced glycolysis, resulting in enhanced expression of pro-inflammatory cytokines such as *Il23a*, *Il6*, and *Il12a* in dendritic cells [13]. Palmitate also enhances oxidative stress-induced TLR2 and TLR4 expression in peripheral blood mononuclear cells. Moreover, in human monocytic cells, palmitate triggers matrix metalloproteinase-9 and chemokine CCL4 production via TLR4/MyD88-dependent signaling to induce subsequent metabolic inflammation [14,15], suggesting a role of innate receptors in sensing metabolic fluctuations [16]. However, whether TLRs serve as CD36 co-receptors for palmitate bioactivity remains unclear. In addition to TLR2 and TLR4, TLR10 has been shown to serve as a mediator in metabolic inflammation. Oxidative stress induces TLR10 expression in adipose tissue in obesity and type 2 diabetes [17].

The NOD-like receptor family, *pyrin domain-containing 3* (NLRP3) inflammasome, is a well-recognized innate receptor system that senses multiple stimuli, molecular patterns, and metabolites, such as palmitate, monosodium urate crystals, and cholesterol crystals, and it has been linked to several inflammatory dysregulations, including metabolic disorders [19,20,21]. Palmitate has been shown to activate NLRP3 and drive inflammation through a lysosome-dependent pathway [22]. It has been demonstrated that macrophages exposed to palmitate in combination with LPS activate lysosome-dependent NLRP3 inflammasome [22]. Mechanistically, the release of lysosomal calcium stores contributes to stabilize the IL-1β transcript via a calcineurin-associated mechanism. On the other hand, lysosome rupture due to palmitate exposure releases cathepsin B, which is required for the activation of the NLRP3-caspase 1 complex for IL-1β secretion [22]. In addition, the crystals formed from saturated fatty acids, including palmitate, have been implicated in lysosome damage-associated NLRP3 inflammasome activation and IL-1β production in macrophages [18]. Likewise, Kalugotla et al. provided evidence showing that the deletion of acyl-CoA synthetase 1 (ACSL1), an enzyme responsible for the esterification of saturated fatty acids, reduces fatty acid crystal formation, thereby decreasing lysosome damage and IL-1β release [23]; this observation suggests the important role of lysosome integrity in the regulation of palmitate-induced inflammasome activation. Moreover, loss of NLRP3 abrogates palmitate-induced IL-1β production in macrophages, suggesting that palmitate regulates NLRP3 inflammasome-mediated cytokine production [24]. Palmitate exposure induces the upregulation of NOD-like receptor C4 (NLRC4), a critical component of the inflammasome complex for IL-1β processing, as well as IL-1β production via TLR4 in tumor-associated macrophages (TAMs) from liver metastases of colorectal cancer. Palmitate stimulation also contributes to M2 polarization of hepatic TAMs, leading to vascular endothelial growth factor (VEGF) production to support tumor growth [25]. Microglia, the primary myeloid cells in the brain, produce IL-1β through palmitate-induced inflammasome activation and ceramide synthesis. Ceramide inhibition diminishes palmitate-induced IL-1β secretion, indicating another layer of regulation for palmitate-involved neuron inflammation [26].

Palmitate-induced lipotoxicity is mainly mediated by overproduction of reactive oxygen species (ROS) and calcium dysregulation. Cellular palmitate is converted to palmitoyl-CoA, which in turn undergoes mitochondrial β-oxidation, a process leading to the generation of ROS [27]. Palmitoyl-CoA also serves as a precursor for ceramides generation, which enhance ROS production through the activation of the mitochondrial respiratory chain (Figure 1) [28]. ROS production also results from PKC activation by DAG (Figure 1), which is synthesized from palmitate-derived phosphatidic acid. Elevated ROS level by palmitate stimulation impairs the endoplasmic reticulum (ER) redox status and results in dysregulation of calcium homeostasis, which results in calcium release from the ER and induction of ER stress to overproduce ROS [29]. The entry of excessive cytosolic calcium into the mitochondria leads to the release of cytochrome C, which subsequently activates the caspase cascade to induce cell death [27]. In contrast, the unsaturated fatty acid oleate prevents palmitate-induced ER stress by the reduction of ROS production [30]. Moreover, oleate can also activate diacylglycerol acyl transferase to convert DAG to triacylglycerol, thus diminishing both the level of DAG and palmitate-derived cytotoxicity [31]. These results suggest that the levels of intracellular palmitate derivatives play pivotal roles in orchestrating palmitate-mediated cell fates. Unbalanced levels of palmitate-derived metabolites may disturb organelle functions such as mitochondria and ER activities, and contribute to lipid-induced cellular responses.

Invariant NKT (iNKT) cells are shown to be involved in autoimmune diseases such as arthritis. Interestingly, palmitate shows an inhibitory effect on iNKT cell activation through inducing *t-bet* and *gata3* mRNA degradation via the IRE1α-dependent pathway to ameliorate arthritis progression [32]. Meanwhile, γδT cells, which are innate-like T lymphocytes, express IL-17A in response to palmitate stimulation and contribute to steatohepatitis progression. Elimination of γδT cells ameliorates fat diet-induced steatohepatitis and facilitates disease resolution [33].

### 2.3. Palmitate Modulates Immune Responses Through Cooperation with Surrounding Cells

**Figure 2:**
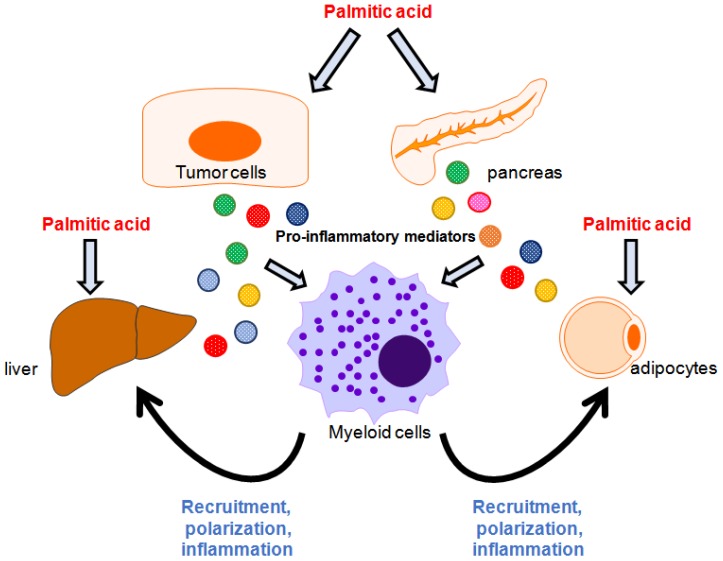
Interplay between myeloid cells and tissues in palmitic acid-induced inflammation. Palmitic acid induces inflammatory responses in the liver, adipocytes, pancreatic islets, and cancer cells to generate pro-inflammatory mediators, leading to the recruitment of, activation of, or phenotypic change in myeloids and creating a patho-immunological environment for disease development.

In addition to stimulating innate immune cells to regulate their inflammatory responses, palmitate has been shown to modulate the behaviors of innate cells through neighboring cells (Figure 2). For example, palmitate upregulates IL-6, IL-8, TLR2, and adhesion molecule ICAM-1 in human adipose microvascular endothelial cells, which in turn promote monocyte recruitment, adhesion, and transmigration [34]. It has been shown that high-fat diet (HFD)-induced inflammation and insulin resistance are tightly regulated by macrophages [35]. Recently, Chen et al. revealed that palmitate induces chemotactic activity and M1 polarization in bone marrow-derived macrophages (BMDMs) through formyl peptide receptor 2 (Fpr2)-mediated pathways, as decreased chemokine and pro-inflammatory cytokine expression has been observed in Fpr2-deficient BMDMs [36]. However, adipocytes receiving palmitate stimulation also secrete pro-inflammatory mediators to modulate phenotypic changes in macrophages. Therefore, crosstalk between macrophages and the surrounding tissues, which are exposed to palmitate, is the critical determinant for the behavior of and phenotypic changes in macrophages [36]. Pancreatic islet inflammation resulting from metabolic stress-induced obesity is tightly associated with dysregulated insulin secretion from β cells. Dalmas et al. demonstrated that palmitate stimulates islet mesenchymal cells to produce IL-33, which in turn activates group 2 innate lymphoid cells that induce retinoic acid secretion by macrophages and dendritic cells in an IL-13-dependent manner. Retinoic acid thereby promotes insulin secretion in β cells [37]. These results suggest that the crosstalk between palmitate-responsive pancreatic islets and local innate cells cooperatively regulates insulin secretion. Hepatocytes undergoing palmitate challenge trigger the production of TLR4 and nuclear factor-κB (NF-κB)-mediated pro-inflammatory regulators through RIP3 and NLRP3 inflammasome activation, leading to hepatic macrophage-associated inflammation; this indicates a pathological role of the lipid metabolite-induced interaction between hepatocytes and macrophages [38]. Additionally, palmitate can trigger lipotoxicity in hepatocytes via activation of the TLR4–IRE1α axis [39]. Lipotoxic hepatocytes secrete extracellular factors, including macrophage chemokine CXCL10, resulting in macrophage recruitment [40]. Accumulated hepatic macrophages exposed to palmitate preferentially skew toward M1 polarization, which is characterized by elevated expression of pro-inflammatory cytokines, such as IL-6 and TNF-α, leading to hepatic inflammation [41,42]. In addition, hepatocyte-derived sphingosine 1-phosphate-containing extracellular vesicles enhance macrophage chemotaxis in palmitate-induced lipotoxic inflammation [43]. Collectively, palmitate-induced lipotoxicity reprograms innate immune cell phenotypes via the stressed tissues; however, the effects of palmitate-induced lethal and sublethal lipotoxic stress on the modulation of immune cell behaviors require further investigation.

### 2.4. Palmitoylation

Post-translational protein modifications tightly control protein quality and function. In addition to phosphorylation, protein palmitoylation is one of the most widely documented protein modifications and plays a pivotal role in regulating protein activity. Palmitoylation often involves the conjugation of palmitic acid to a cysteine residue in a polypeptide chain. In immune cells, palmitoylation regulates the surface receptor trafficking and coordination involved in the relevant signaling pathways [44]. For example, cytosolic domain palmitoylation of P2X7, a purinergic receptor for extracellular ATP signal transduction, prevents receptor desensitization and reveals the unique properties of P2X7 signals [45]. P2X7 receptors are highly expressed membrane proteins in innate immune cells, particularly in mast cells, monocytes, macrophages, and microglia. Upon P2X7 receptor activation via ATP, pro-inflammatory cytokines such as IL-1β and IL-18 are released through cooperation with the inflammasome and activation of other innate receptors [46,47]. Additionally, strong activation of the P2X7 signaling pathway contributes to membrane permeabilization and cell death in macrophages and is associated with pathological inflammation [48].

In addition to the regulation of cell surface localization by protein palmitoylation, palmitoylated proteins target intracellular organelles. Yang et al. demonstrated that following geranylgeranylation, Rac1 palmitoylation promotes its translocation into mitochondria-associated endoplasmic reticulum (ER) membranes, which are junctions between mitochondria and the ER membrane, in response to viral infection [49]. Mitochondria-associated ER membrane-localized Rac1 shows an inhibitory effect on the activation of mitochondrial antiviral signaling protein, thereby limiting virus-induced innate immune response [49]. Estrogen receptor (ER36) is shown to be responsible for 17-β-estradiol-mediated anti-inflammatory activities in monocytes and macrophages [50]. Notably, ER36 palmitoylation in breast cancer cells, but not immune cells, promotes nuclear membrane localization [51], highlighting a potential role of palmitoylation in steroid-mediated innate immune regulation. Stimulator of interferon genes (STING) protein is a cytosolic DNA-sensing receptor involved in triggering front-line immune response. A recent study showed that STING palmitoylation is required for its multimeric assembly at the Golgi apparatus, which in turn recruits downstream signaling components to mediate inflammatory cytokine production [52]. Inhibiting STING palmitoylation using small molecule compounds reduces STING-triggered inflammatory cytokine production in macrophages, suggesting an essential role of palmitoylated STING in innate immune response.

Recently, an interferon-responsive protein, IFN-induced transmembrane protein 2 (IFITM2), was reported to be shuttled from hepatocytes to dendritic cells (DCs) by exosomes to inhibit type I interferon induction in DCs. Interestingly, inhibition of IFITM2 palmitoylation by an inhibitor or genetic mutations in the palmitoylated cysteine residues reduces exosomal IFITM2 incorporation and relieves the suppression of DC-induced innate immune response, therefore adding another layer of complexity to the regulation of palmitoylation on innate immune response through intercellular communication [53].

## 3. Palmitate in Metabolic Disorders

### 3.1. Palmitate in Diabetes

Diabetes development often results from obesity that is associated with low-grade, long-lasting inflammation. The level of circulating saturated fatty acids, particularly palmitate, is elevated and is responsible for triggering an inflammatory response, which induces infiltration of macrophages, thereby promoting diabetic inflammation. Extracellular palmitate induces inflammation through the transduction of various cellular pathways. For example, in human monocyte-derived macrophages, palmitate was implicated in ER stress induction and NLRP3 inflammasome activation to produce IL-1β via a caspase 1-dependent mechanism. Elevated IL-1β secretion is correlated with increased macrophage infiltration in pancreatic islets and type 2 diabetes [54,55]. In addition to inducing inflammasome activation, palmitate can promote IL-1β and IL-18 production through caspase 4/5-mediated inflammatory cell death, known as pyroptosis, in human monocytes [56]. Pro-inflammatory cytokines such as IL-6 and TNF-α are also involved in the development of palmitate-induced inflammation. Upon palmitate stimulation, peritoneal macrophages induce IL-6 and TNF-α release via JNK-mediated pathways [57]. JNK deletion in macrophages reduces infiltrating macrophages and protects mice against obesity-associated insulin resistance and type 2 diabetes [58]. In an HFD-induced obese mice model, palmitate aggravated proteinuria-induced cell death and inflammation via the CD36–NLRP3–caspase-1 axis in kidney renal tubular cells [59]. CD36 inhibition by sulfo-N-succinimidyl oleate attenuated HFD-associated renal inflammation and injury [59].

In contrast, free unsaturated fatty acids such as oleate can alleviate palmitate-induced toxicity. Indeed, oleate has been demonstrated to sequester palmitate into triglyceride pools to prevent palmitate-induced cell death [60]. Additionally, oleate also protects palmitate-induced β-cell apoptosis by suppression of unfolded protein response-induced ER stress [61]. Moreover, other unsaturated fatty acids such as linoleate also diminish IL-1β release induced by palmitate and other NLRP3 inducers [62]. These findings suggest an anti-inflammatory mechanism followed by unsaturated fatty acids to inhibit the palmitate-triggered diabetic inflammatory pathway. In addition to extracellular palmitate-induced cellular response, intracellular palmitate synthesis from de novo lipogenesis, which is mediated by fatty acid synthase (FAS), also plays a critical role in controlling macrophage function [63]. Wei and colleagues reported that FAS-mediated endogenous palmitate synthesis is required for membrane composition and configuration in macrophages and affects macrophage-induced chronic inflammation [64]. FAS deletion in macrophages inhibits diet-induced macrophage recruitment, chronic inflammation, and insulin resistance, thereby preventing diabetic inflammation [64]. Endogenous palmitate can also be shifted toward triglyceride storage to prevent its damaging effects via the cellular desaturase enzyme stearoyl-CoA desaturase (SCD) [60]. Knockdown of SCD in β cells lowers desaturation of palmitate, resulting in an increased susceptibility of β cells to palmitate-induced ER stress and apoptosis [65]. Collectively, stimulation of saturated fatty acid palmitate renders cells more susceptible to cytotoxicity. However, unsaturated fatty acids such as oleate and linoleate exhibit the beneficial effect of countering palmitate-induced cellular toxicity. They may maintain the balance between the pools of saturated and unsaturated fatty acids. Thus, it is important to further investigate the interplay between these two lipid species in physiological and pathological conditions. It is also important to examine how their transport, biosynthesis, and modifications orchestrate lipotoxicity and inflammation in lipid metabolic disorders such as diabetes.

### 3.2. Non-Alcoholic Fatty Liver Disease (NAFLD)

Non-alcoholic fatty liver is characterized by increased fat deposition in hepatocytes and is presently a prevalent health problem globally. NAFLD is often associated with type 2 diabetes, metabolic inflammation, and HFD-induced obesity. Palmitic acid is the major component in high-fat Western-style diets and induces hepatocyte fat deposition. Moreover, lipid accumulation in hepatocytes induces hepatic inflammation and results in hepatocellular injury and fibrosis, thereby leading to cirrhosis and hepatocellular carcinoma development. Recently, using conditional STING knockout in myeloid cells, Luo et al. demonstrated that dietary palmitate induces STING activation in macrophages to produce pro-inflammatory cytokines, which in turn promote hepatic fat deposition and contribute to the development of non-alcoholic fatty liver disease [66]. A subgroup of patients with non-alcoholic fatty liver disease can develop advanced hepatitis and liver damage, which are characteristics of non-alcoholic hepatic steatosis (NASH). This population is correlated with an increased risk of cirrhosis, hepatocellular carcinoma, and liver failure [67]. Palmitate-induced hepatocyte apoptosis has been demonstrated to be mediated mainly by death receptor TRAIL-R2 [68]. Moreover, patients with NASH show increased TRAIL-R2 expression in the liver [69]. Similar results were also observed in a NASH mouse model [70]. However, loss of TRAIL-R2 abrogates palmitate-induced hepatocyte cell death [68]. Caspase 8 serves as a critical mediator for the transduction of TRAIL-R2-dependent lipoapoptosis signaling. Active caspase 8 promotes mitochondrial cytochrome c release and cell apoptosis via ER stress and TRAIL-R2-mediated pathways [71]. Lipotoxic hepatocytes increase sonic hedgehog secretion and upregulate hepatic osteopontin production in an autocrine fashion; elevated osteopontin therefore promotes macrophage-induced pro-inflammatory response. Hedgehog pathway inhibition by pharmacological inhibitors suppresses hepatic inflammation and prevents NASH development [70,72]. Emerging evidence has shown that hepatocytes receiving palmitate stimulation promote extracellular vesicle release, which in turn induces macrophage chemotaxis and the expression of pro-inflammatory cytokines, including IL-6 and IL-1β, in the NASH animal model [40,73].

## 4. Involvement of Palmitate in Immune-Related Cell Interactions during Cancer Development

Dysregulation of lipid metabolism has been linked to tumorigenesis. It has been shown that palmitate induces CCL4 expression through TLR4-mediated pathways in monocytes [15]. Interestingly, CCL4 expression plays an important role in the migration of tumor-infiltrating lymphocytes with immunological regulatory phenotypes in melanoma [74]. Recently, palmitate and TNF-α were shown to synergistically promote CCL2 production in monocytes [75], and CCL2 was demonstrated to drive radioresistance, metastasis, and epithelial–mesenchymal transition in nasopharyngeal carcinoma [76]. Additionally, palmitate induces monocytes to produce macrophage inflammatory protein 1 [77], a chemokine responsible for monocyte trafficking into tumor regions [78]. Collectively, these findings suggest that palmitate regulates tumor progression, at least in part, by modulating innate immune cell movement. However, the crosstalk between palmitate-triggered metabolic reprogramming in immune cells and tumor progression remains unclear.

ACSL1 is an enzyme that modifies palmitate, upon which the latter initiates an intracellular downstream signaling. Palmitate-treated bone marrow-derived macrophages (BMDMs) displayed “M1-like” proinflammatory polarization, which was characterized by an elevated expression of genes including *Tnf*, *Cxcl1*, *Il6*, and *Nos2* [79]. Moreover, the TNF-α-induced pro-inflammatory phenotypic shift in monocytes is dependent on ASL1 activity, as inhibition of ACSL1 activity by an inhibitor abolished TNF-α-activated metabolic inflammation [80]. Recently, in breast cancer cells, ACSL1 has been reported to regulate TNF-α-induced production of granulocyte–macrophage colony-stimulating factor (GM-CSF) [81], an essential growth factor for myeloid cell differentiation. Interestingly, in response to palmitate, selective deletion of ACSL1 in myeloid cells reduces lysosome-dependent NLRP3 inflammasome activation and the production of IL-1β [23], an inflammatory cytokine that is tightly associated with tumor progression, suggesting the role of palmitate in cancer development. Although emerging evidence shows the correlation between palmitate-related inflammation and cancer development, the mechanism by which palmitate modulates the activity and/or immunological phenotypes of innate immune cells in the tumor microenvironment during tumor development remains to be further elucidated.

CD36 serves as the surface receptor for palmitate uptake and plays a critical role in palmitate-induced signaling pathways. It has been shown that increased CD36 expression in gastric cancer is correlated with poor prognosis [82]; accordingly, palmitate promotes gastric cancer metastasis in a CD36-dependent manner [83]. Palmitate stimulation increases monocytic CD36 expression, macrophage differentiation, and proinflammatory cytokine production [84]. Recently, Kitamura et al. showed that precursor cells of metastasis-associated macrophages (MAMs), a cell type harboring immunosuppressive characteristics for metastasis promotion, express CD36 levels comparable to those of MAMs [85]; this implies a potential role of palmitate in MAM differentiation from its progenitors, thereby promoting tumor development.

## 5. Targeting of Palmitate-Induced Pathways as a Therapeutic Strategy

Since CD36 serves as the major transporter for cell palmitate uptake, the targeting of palmitate-mediated signaling has emerged as a potential therapeutic tactic. Indeed, palmitate-induced metastasis of gastric cancer is dependent on CD36, and high CD36 expression levels in gastric cancer patients correlate with poor clinical outcome; therefore, there is a possibility that blocking of the CD36–palmitate axis may serve as a potential gastric cancer treatment [83]. Indeed, CD36 deletion in a prostate cancer animal model revealed a dramatic decrease in fatty acid uptake and ameliorated cancer progression [86]. Immune cells play pivotal roles in regulating tumor progression. CD36 is responsible for lipid uptake to trigger downstream inflammatory signaling in innate immune cells, and it has been reported that lipid deposition in dendritic cells inhibits antigen cross-presentation and dampens anti-tumor immune response [87]. Therefore, CD36 may be a potential target for cancer treatment. Although several preclinical studies and clinical trials targeting CD36 for cancer treatment are being performed [88], further studies are needed to explore the effects of CD36 on innate immune cell-mediated anti-tumor functions. It has been shown that palmitoylation of CD36 enhances fatty acid uptake activity and stabilizes its plasma membrane translocation [89]. Patients with NASH show increased CD36 palmitoylation, which causes lipid accumulation and enhanced inflammatory signaling [90]. However, the effects of CD36 palmitoylation targeting on palmitate-induced inflammation require further investigation.

In addition to blocking palmitate entry by CD36 inhibitors, molecules that disturb intracellular palmitate modification and the associated pathways are also potential targets. For example, ACSL1 is one of the best-studied acyl-CoA transferases, and it can be induced by TLR- and inflammatory mediator-mediated signaling. ACSL1 catalyzes the conversion of several fatty acids to fatty acyl-CoAs, which enables their intracellular activity. Deletion of ACSL1 in macrophages results in reduced palmitate crystals and subsequent inflammasome-induced IL-1β release [23], suggesting that ACSL1-dependent conversion of palmitate to palmitoyl-CoA plays a critical role in palmitate-induced crystallization and contributes to lipotoxic inflammasome activation. In addition, myeloid cell-specific deletion of ACSL1 in a diabetic mouse model prevented the formation of the inflammatory phenotype of macrophages, which is associated with diabetes [91]. Therefore, ACSL1 acts as an important player in immunometabolism [92]. Thus, pharmacological inhibition of ACSL1 may provide promising therapeutic value in treating inflammatory-associated disorders such as cancer [93].

FAS is critical for de novo palmitate production. FAS targeting by orlistat, a small molecule originally approved by the Food and Drug Administration (FDA) for obesity management, reveals a promising approach for cancer treatment [94]. Recently, Kant et al. reported that in tumor-bearing mice, orlistat administration skewed macrophage polarization toward an anti-tumor M1 phenotype, as characterized by the generation of reactive oxygen species, elevated phagocytosis, and enhanced tumoricidal activity [95]. Taken together, these findings suggest that targeting FAS may modulate immune cell phenotypes, leading to inhibited disease progression.

## 6. Concluding Remarks

Palmitate functions in various pathological cell processes, including inflammatory diseases, metabolic disorders, and tumor progression. Palmitate-induced metabolic reprogramming in innate immune cells modulates inflammatory responses and contributes to disease progression. Further investigation will be needed to better understand the full range of palmitate functions in the front-line immune response of the host, as well as to decipher the pathological role of palmitate. In addition, palmitate-mediated pathways are emerging as potential target candidates for therapeutic intervention in inflammatory-associated diseases. Moreover, selectively targeting the specific downstream effector of the palmitate-mediated pathway that triggers pathological inflammation is required, as palmitate uptake may control cellular lipid homeostasis in physiological conditions.
